# Inhibitory Effect of Amentoflavone on the Virulence of MRSA by Targeting ClpP

**DOI:** 10.1111/jcmm.70825

**Published:** 2025-10-22

**Authors:** Teri Gele, Xiangri Kong, Qiuyue Zhang, Wu Song, Junpeng Guo, Paizati Hamidi, Kulishasi Mani, Shaoyu Han, Xuan Zhao, Jingwen Chen, Chi Zhang, Abduldayeva Aigul Abduldayevna, Qingjie Li

**Affiliations:** ^1^ Changchun University of Chinese Medicine Changchun China; ^2^ Endocrinology Department The Affiliated Hospital of Changchun University of Chinese Medicine Changchun China; ^3^ Research Center of Traditional Chinese Medicine The Affiliated Hospital to Changchun University of Chinese Medicine Changchun China; ^4^ Xinjiang Altay Regional Hospital of Traditional Chinese Medicine Altay China; ^5^ The University of Queensland St Lucia Queensland Australia; ^6^ Astana Medical University Astana Kazakhstan

**Keywords:** antivirulence strategies, ClpP, MRSA, natural compound

## Abstract

Methicillin‐resistant *Staphylococcus aureus* (MRSA) poses significant therapeutic challenges due to its global spread and virulence. Targeting the critical virulence regulator ClpP presents a promising antivirulence strategy. This study investigated AMF's mechanism against MRSA through molecular dynamics simulations, FRET and TSA. Phenotypic analyses revealed AMF's inhibition of MRSA haemolytic activity (72% reduction) and biofilm formation (58% decrease) without affecting bacterial growth. Molecular docking identified key AMF–ClpP interaction sites (ARG‐171, ASP‐170, ASP‐172), validated via CETSA. AMF reduced transcription of critical virulence genes (*hla, psmα*) by 3.8‐fold and inhibited ClpP enzymatic activity by 65%. Cellular studies demonstrated AMF's protection of A549 lung cells from MRSA infection (82% viability vs. 43% control). In murine pneumonia models, AMF treatment enhanced survival rates from 20% to 75% while reducing proinflammatory cytokines (IL‐6, TNF‐α) by 60%–70%. Histopathological analysis showed significant mitigation of lung tissue damage. These findings establish AMF as a potent ClpP inhibitor that attenuates MRSA virulence through dual mechanisms: suppression of toxin production and biofilm formation. The compound's therapeutic potential stems from its ability to disarm pathogenic mechanisms while maintaining commensal microbiota integrity. This study provides proof‐of‐concept for antivirulence strategies targeting ClpP, offering a promising alternative to traditional antibiotics against MRSA infections.

**Abbreviations**
Agr: accessory gene regulator
ATCC: American Type Culture Collection
CETSA: cellular thermal shift assay
ClpP: caseinolytic protease P
CLSI: Clinical and Laboratory Standards Institute
*E. coli*
: *Escherichia coli*
FRET: fluorescence resonance energy transfer
H&E: haematoxylin–eosin staining
HA‐MRSA: hospital‐acquired methicillin‐resistant *Staphylococcus aureus*
Hla: α‐hemolysin
IPTG: isopropyl‐β‐d‐thiogalactoside
ka: association rate constant
KD: affinity constant
kd: dissociation rate constant
MIC: minimal inhibitory concentration
MRSA: methicillin‐resistant *Staphylococcus aureus*
PVL: Panton–Valentine leucocidin
RMSD: root mean square deviation
RMSF: root‐mean‐square fluctuation
*S. aureus*
: *Staphylococcus aureus*
SASA: solvent‐accessible surface area
TSA: thermal shift assay
ΔGbind: total binding free energy

## 1 Introduction

The incidence of *Staphylococcus aureus* infection peaks among children and adolescents under 17 years of age [1]. When influenza is present, the mortality rate from *S. aureus* ‐induced pneumonia, a life‐threatening lung infection, can rise to 50% [2]. Methicillin‐resistant *S. aureus* (MRSA) has been designated as a “priority pathogen” by the World Health Organization due to its extensive prevalence and the severe nature of the infections it induces. It could lead to serious and infectious diseases such as pneumonia, sepsis and toxic shock syndrome (TSS) [3]. These bacteria often show resistance to multiple antibiotics, complicating treatment and effective therapies remain limited. Overall MRSA infection rates have risen significantly over the past three decades, with different MRSA prevalence rates varying in different regions. For HA‐MRSA prevalence, it is 19.1% in Mexico [4] in 2021, 34.8% in Nigeria [5] and 15.1% in Australia [6] in 2020; while the prevalence of another category, CA‐MRSA, reaches 44.3% in India during 2019–2020 [7]. MRSA has long been acknowledged as a critical nosocomial pathogen, responsible for infections in healthcare settings. The emergence of new strains, like healthcare‐associated MRSA (HA‐MRSA), has become a major concern in developed countries, posing significant threats of pneumonia, skin infections and bloodstream infections [8]. The emergence of MRSA as a major cause of severe infections has placed significant strain on healthcare facilities and public health systems [9]. Recent advancements in antivirulence therapies have demonstrated potential in managing MRSA infections. Consequently, mitigating the virulence of MRSA could serve as an effective strategy for infection control.

ClpP, a proteolytic enzyme, degrades misfolded proteins and plays a crucial role in the synthesis and control of numerous virulence proteins in *S. aureus* , including alpha‐hemolysin (Hla), leukocidin (PVL), surface protein A (SpA) and phenol‐soluble module A [10]. Hla is a crucial virulence factor that enhances bacterial pathogenicity through the formation of pores in target cell membranes, disrupting solute balance and leading to erythrocyte lysis [11]. PVL specifically affects human neutrophils and monocytes, disrupting solute balance and inducing inflammatory responses. Research elucidated that ClpP plays a pivotal role in the intricate processes of biofilm formation and bacterial adhesion to host cells [12]. The activation of ClpP triggers unrestrained hydrolysis of *S. aureus* peptide chains, culminating in bacterial death. Conversely, the inhibition of ClpP reduces pathogenicity and enhances the host immune system's ability to clear. This is consistent with antivirulence strategies. Given its crucial role in virulence proteins and biofilm formation in *S. aureus* , ClpP stands out as an exceptionally promising target. Virulence factors regulated by ClpP are integral to various stages of bacterial infection in the host [10, 11, 12, 13]. As a representative of a highly effective antibacterial target, ClpP exerts significant regulatory control over the virulence of diverse pathogenic bacteria.

Amentoflavone (AMF), a natural flavonoid compound, exhibits unique biological activity owing to its chemical structure, characterised by a bis‐arene structure composed of two benzene rings connected through oxygen atoms. Extensive research has illuminated the multifaceted pharmacological potential of AMF, such as antioxidant [14], anti‐inflammatory [15], antimalignant tumour [16, 17], antibacterial [18] and antiviral [19] effects.

Our study has unveiled that AMF directly interacted with ClpP, thereby suppressing its activity in *S. aureus* , which in turn curtails the virulence of MRSA and hinders its biofilm formation. AMF counteracted the damaging effects of *S. aureus* on red blood cells. In vivo studies have corroborated the protective efficacy of AMF against lethal pneumonia instigated by MRSA. These insights provided a preliminary elucidation of the mechanism through which AMF attenuated the virulence of MRSA, paving the way for potential therapeutic interventions for MRSA‐induced infections.

## 2 Materials and Methods

### 2.1 Bacteria, Gene Vectors, Culture Methods and Materials

In this study, *Escherichia coli* ( *E. coli* ) and *S. aureus* were inoculated in Luria–Bertani (LB) medium or trypticase soy broth (TSB) at 37°C. AMF was purchased from the original company (purity ≥ 98%, Yeasen, Shanghai, China) and dissolved in Dimethyl sulfoxide to obtain a 20 mg/mL solution. *S. aureus* USA300 strain number (ATCC BAA‐1717) was obtained from the American Type Culture Collection (ATCC, Virginia, USA). The *E. coli* BL21(DE3) pET28a::*clpP* strain and *S. aureus* Δ*clpP* mutant strain were originally maintained by our laboratory. TSB, brain heart infusion (BHI) agar and CAMHB stock were purchased from Qingdao Hope Biotechnology Company, China. The experiment used A549 human lung cancer cells (ATCC, VA, USA) and fluorescent peptides (Sigma‐Aldrich, STL, USA). The Biosafety Committee of Changchun University of Traditional Chinese Medicine approved this study.

### 2.2 Extract Eluted ClpP Protein and Examine

*E. coli* BL21 (DE3) strain carrying episome pET28a‐clpP was introduced into LB liquid medium broth supplemented with kanamycin at a concentration of 50 μg/mL. The culture was subsequently grown to an optical density of OD_600_ = 0.8 at a temperature of 37°C and a speed of 220 rpm (rpm). Isopropyl‐β‐d‐thiogalactopyranoside (IPTG, Vicki, Sichuan, China) was added to the bacterial culture when OD_600_ = 0.6. The °C and 180 rpm for 16 h. The bacterial pellet should be collected and then mixed with lysis buffer containing 50 mM Tris, 100 mM NaCl and a pH of 8.5. After quickly mixing, the sample should be resuspended and lysed using low‐temperature sonication. The resuspended mixture was centrifuged at 18,000 rpm for 50–60 min at low temperature. The supernatant was then collected and transferred into a His‐Trap column. Detection of ClpP protein band by western blot analysis, the system was once more utilised to express and purify mutant ClpP proteins.

### 2.3 Molecular Docking

The ClpP three‐dimensional model was accurately retrieved from the RCSB Protein Data Bank, with specific IDs being 3V5E, 4MXI, 4EMM and 3TS9 configurations. The three‐dimensional model of AMF was simulated using ChemBio3D Ultra 12.0 software for further analysis. AutoDock Vina 1.1.2 software was used to predict molecular docking interactions and determine reasonable conformations for the docking process.

### 2.4 Molecular Dynamics Simulation

These molecular dynamics simulations use Gromacs2022.3 version software for molecular dynamics simulation [20]. The standard parameters of static temperature 300 K and pressure 1 Bar were inputted into Gromacs2022.3 using the force field Amber99sb‐ildn and the solvent consisting of water molecules (Tip3p water model). The number of Na^+^ ions was controlled to neutralise the total charge of the simulation system. The steepest descent method is utilised in Gromacs version 2022.3 to minimise energy. Subsequently, 100,000 steps of isothermal–isovolumetric ensemble (NVT) equilibrium and isothermal–isobaric ensemble (NPT) equilibrium are conducted, employing a coupling constant of 0.1 ps over a duration of 100 ps. Finally, a free molecular dynamics simulation was run, with a total of 5,000,000 steps, a step size of 2 fs and a total time of 100 ns. After analysing the trajectory using the software's tools, we calculated the root mean square deviation (RMSD), root mean square fluctuation (RMSF) and protein gyration radius. Additionally, we obtained free energy profiles and other relevant data [21].

### 2.5 Determination of the Minimal Inhibitory Concentration (MIC)

Briefly, 5 × 10^4^ CFU/mL *S. aureus* USA300 was inoculated into a 96‐well plate. Serially diluted AMF (0–512 μg/mL) was then added, along with vancomycin (2 μg/mL final) as a positive control in designated wells, to observe the growth of MRSA and obtain the MIC of AMF.

### 2.6 ClpP Activity Assay

ClpP activity was determined using the fluorescence resonance energy transfer (FRET) method. Briefly, a mixture of ClpP buffer (80 mM HEPES, 80 mM NaCl, pH = 7.0), ClpP protein (2 μM) and different amounts of AMF (0.5 ~ 512 μg/mL) was added to a 96‐well plate (SORFA, China) and allowed to stand for 1 h. The fluorescent peptide substrate Suc‐LY‐AMC (Sigma‐Aldrich, STL, USA) was injected with a pipette and reacted at 37°C for 0.5 h. The solution system consisted of ClpP buffer, ClpP protein (2 μM), AMF gradient (from 0.5 to 512 μg/mL), fluorescent peptide substrate and an equal amount of DMSO as a negative control. Fluorescence intensity was measured using a microplate reader with emission and excitation wavelengths set at 465 and 360 nm, respectively.

### 2.7 Thermal Shift Assay (TSA)

TSA methods effectively identify protein interactions with small molecules, ligands or drugs. Target protein (1 μM), Sypro Orange (Sigma‐Aldrich, Germany), AMF (64–512 μg/mL), 130 mM NaCl and 10 mM HEPES were injected into the PCR plate at pH = 7.5. A real‐time PCR machine was utilised to heat the PCR plate from 20°C to 85°C at a heating rate of 2°C/min. The confirmation of this process was validated by monitoring changes in fluorescence intensity over time following the addition of the drug. The temperature at which half of the protein is denatured is commonly referred to as the *T*_m_ value.

### 2.8 Cellular Thermal Shift Assay (CETSA)

*E. coli* BL21(DE3) pET28a::*clpP* was grown until OD_600_ = 0.8, and 0.5 mM IPTG was added dropwise. The separated liquid was allowed to stand at 37°C for 1 h with AMF and an equal amount of dimethyl sulfoxide, and then centrifuged at 15,000 *g* for 30 min at low temperature. The reaction was heated in a gradient between 25.0°C and 65.0°C for 7 min. The mixture was then immediately immersed in low heat and allowed to stand for 3 min. Subsequently, the supernatant was centrifuged at 15,000 g for 30 min, after which it was collected and subjected to western blot. Finally, the relative intensity of protein expression was quantified using ImageJ software (NIH).

### 2.9 Urease Activity

AMF (64–512 μg/mL) was added to the TSB medium of USA300 and USA300‐ΔclpP, and cultured from OD_600_ = 0.3 until OD_600_ = 2.5. AMF‐treated MRSA and DMSO‐treated MRSA with TSB medium were then inoculated onto urease agar containing phenolsulfonphthalein and incubated at 37°C for 1 day. The ClpP mutant strain was used as a control group. When urease catalysis is no longer blocked, the indicator turns red. The temperature was raised to melt the medium and then the OD_450–600_ full wavelength photometric values were measured using an enzyme marker (Multiskan Go, USA).

### 2.10 Quantitative Real‐Time PCR (RT‐qPCR)

In accordance with the instructions of the kit, Trizol was utilised to extract total RNA from *S. aureus*. The concentration of total RNA was determined by UV spectrophotometer and confirmed to meet the required standards. The purified and enriched *S. aureus* RNA was reverse transcribed into bacterial cDNA using Transcriptor First Strand cDNA Synthesis Master Mix (5X) (Thermo Fisher, USA). RT‐qPCR analysis was performed using Fast Start Universal SYBR green Master (Beyotime, Shanghai, China) staining and CFX96 Touch real‐time PCR detection system (Bio‐Rad, USA). The reference gene is *16sRNA*. Three independent experiments were performed, and the PCR primer sequences are shown in Table 1.

**TABLE 1:** Primers used in RT‐qPCR.

Primer name	Oligonucleotide
16S‐F	5′ TGATCCTGGCTCAGGATGA 3′
16 s‐R	5′ TTCGCTCGACTTGCATGTA 3
hla‐F	5′ GGTATATGGCAATCAACTT 3
hla‐R	5′ CTCGTTCGTATATTACATCTAT 3′
RNAIII‐F	5′ AATTAGCAAGTGAGTAACATTTGCTAGT 3
RNAIII‐R	5′ GATGTTGTTTACGATAGCTTACATGC 3′
pvl‐F	5′ CACAAAATGCCAGTGTTATCCA 3
pvl‐R	5′ TTTGCAGCGTTTTGTTTTCG 3′
Spa‐F	5′ CAGCAAACCATGCTA 3′
spa‐R	5′ GCTAATGATAATCCAAATACAGTTG 3
psmα‐F	5′ TAAGCTTAATCGAACAATTC 3′
psmα‐R	5′ CCCCTTCAAATA‐AGATGTTCATATC 3′
agrA‐F	5′ GCAGTAATTCAGTGTATGTTCA 3′
agrA‐R	5′ TATGGCGATTGACGACAA 3′
ClpP‐F	5′ AACAACAAACCGCGGTGAAC 3′
ClpP‐R	5′ CCATTGATGCAGCCATACCG 3′

### 2.11 Western Blot Analysis

AMF at a concentration of 64–512 μg/mL was added to *S. aureus* USA300 and incubated together for 16 h. The mixture was centrifuged at 5000 rpm for 5 min at 4°C and the supernatant was collected in the EP tube. After sodium dodecyl sulphate polyacrylamide gel electrophoresis, rabbit anti‐staphylococcal Hla antibody (1:5000) (Cell Signaling Technology, Danvers, MA, USA) and HRP‐conjugated goat anti‐rabbit IgG (1:10, iofilm formation was initiated by ad000) (Santa Cruz Biotechnology, Boston, USA). PVL was visualised using rabbit polyclonal anti‐PVL LukS subunit (0.5 mg/mL, Thermo Fisher Scientific, Waltham, MA, USA). All proteins of *S. aureus* were extracted with 12 mg/mL lysozyme, 5 mg/mL staphylococcal lysostaphin, and 250 μL RIPA. Determine ClpP concentration. ClpP binding was performed using rabbit anti‐ClpP polyclonal antibody (1:400, Laboratory homemade). ECL Plus (Yuanye, Shanghai, China) was used to visualise the bands.

### 2.12 Growth Curve

*S. aureus USA300* and Δ*clpP* strains were cultured for 12 h and then inoculated into TSB at higher dilution*s*. The bacteria were grown until OD_600_ = 0.3. Three groups were set up, including the DMSO control group, the AMF (512 μg/mL) drug group and Δ*clpP* group. Detect the OD value of bacterial samples (100 μL) at a wavelength of 600 nm at different time periods.

### 2.13 Haemolysis Assay

When *S. aureus* USA300 and USA300‐Δ*clpP* reached an optical density of 0.3 at 37°C after 16 h in the culture medium, 64–512 μg/mL of AMF was administered and monitored until the optical density of USA300 reaches 2.5.1 mL of supernatant (10,000 rpm, 10 min) was collected, 100 mL of supernatant was incubated with rabbit red blood cells in PBS with a final concentration of 2.5% for 30 min at 37°C, and then centrifuged (8000 rpm, 4°C, 5 min). In addition, the negative group of the experiment used PBS instead of the supernatant, and the positive group used 1% Triton X‐100 instead of the supernatant as a positive control group. The optical density at 543 nm (OD_543_) of the supernatant was determined. To investigate the real‐time neutralising activity of AMF on Hla, various concentrations of AMF (64–512 μg/mL) were diluted in rabbit red blood cells suspended in PBS and subsequently mixed with the bacterial supernatant, incubated at 37°C for 30 min, followed by the measurement of the OD_543_ value.

### 2.14 Biofilm Formation

Biofilm formation was initiated by adding 100 μL of 20% freeze‐dried rabbit blood to 96‐well plates at 4°C for12–16 h. *S. aureus* USA300 or Δ*clpP* were then diluted at a ratio of 1:100 and cultured in BHI containing 1% glucose. Subsequently, the bacterial solution in the 96‐well plates was replaced with 100 μL of bacterial solution per well containing different concentrations of AMF (ranging from 64 to 512 μg/mL). The 96‐well plates were incubated for 24 h and then washed to remove unbound bacteria. Biofilm formation was assessed by staining with 0.1% crystal violet for 20 min, rinsing with sterile PBS, and air‐drying at room temperature. To quantify biofilm formation, 200 μL of 95% ethanol was added to each well, and the absorbance at 595 nm was measured.

### 2.15 Live/Dead Cell Counting and Lactate Dehydrogenase Assay

The overnight‐cultured *S. aureus* was transferred to TSB, it was diluted to the ratio 1:150, and culturing was continued until the OD_600_ reaches 0.5. After centrifugation, the bacteria were washed twice with PBS, and we mixed the bacteria into DMEM. Various gradients of AMF (64–512 μg/mL) were then added to the bacterial solution. The culture medium was aspirated from the 12‐well plate, it was washed three times with phosphate buffer, and then 400 μL of the bacterial solution mentioned above was added. After incubation at 37°C for 5 h, the Live & Dead Calcein AM/PI Cytotoxicity Assay Kit (Carlsbad, CA, USA) was utilised to follow the provided instructions and analyse the results. This procedure aimed to evaluate the potential of AMF to mitigate the damage caused by MRSA on cells. To detect the inhibitory effect of AMF on MRSA‐induced damage to A549 cells, the supernatant was aspirated and the LDH content in the supernatant was measured using an LDH cytotoxicity assay kit to read the product introduction.

### 2.16 Cytotoxicity Assay

The cytotoxicity of AMF against A549 cells was assessed using the MTT method, which involves the use of 3‐[4,5‐dimethylthiazol‐2‐yl]‐2,5‐diphenyltetrazolium bromide. A549 cells were plated in a 96‐well plate at a density of 2 × 10^4^ cells per well and incubated. Various concentrations of AMF (64–512 μg/mL) were added to the plate, followed by a 24‐h incubation period. Subsequently, 10 μL of MTT solution was added, followed by the removal of the liquid after 4 h and the recording of the OD_490_ value.

### 2.17 Pneumonia Model

All animal experiments conducted in this research adhered to the guidelines set by the Experimental Animal Ethics Committee of Changchun University of Traditional Chinese Medicine. To investigate the efficacy of AMF in treating acute MRSA‐induced pneumonia, *BALB/c* male mice (7 weeks old, SPF grade) from Cavens Biogle Biological Company were chosen as the model for pneumonia infection. The mice were kept in a clean, ventilated environment that complied with experimental standards and were supplied with sufficient food and water. For assessing the survival rate, a syringe was used to administer 2 × 10^8^ CFUs of *S. aureus* into the nasal cavity of each mouse, ensuring the bacteria reached the lungs by having the mice stand for 30 s (*n* = 10). Subsequently, the mice were treated with AMF (100 mg/kg·day^−1^), vancomycin (100 mg/kg·day^−1^) or a combination of both (100 mg/kg·day^−1^ each). Perfusion was carried out every 12 h, and the survival rates over 96 h were analysed for each group. Statistical significance of the survival rate curves between the *S. aureus* group, treatment group and blank group was determined using the log‐rank test (*p < 0.01, ***p < 0.001).

Each group of mice received an intranasal instillation of 30 μL (1 × 10^8^ CFU) of *S. aureus* culture for 2 days (*n* = 5) to assess the bacterial load and lung tissue histopathology. Following cervical dislocation at 48 h, the lungs were weighed, homogenised, diluted and plated on BHI agar. Incubation at 37°C overnight allowed for colony counting. The same experimental protocol was used to evaluate pathological examination scores and inflammatory factors. To determine levels of IFN‐γ, IL‐6 and TNF‐α, the trachea of anaesthetised mice was isolated 48 h post‐infection and ligated at the distal end. Alveolar fluid was then collected by PBS lavage (0.5 mL, three times) (*n* = 5). Cytokines were quantified using ELISA kits from eBioscience. The lungs were perfused, fixed in formalin and subjected to H&E staining for histopathological assessment under a light microscope. Inflammation severity was evaluated based on criteria such as inflammatory cell count, interstitial inflammation distribution and injury area (0–2 points for each criterion). Assessment also included edema, effusion and haemorrhage severity (0–5 points each), totaling 26 points.

### 2.18 Statistical Analysis

The statistical significance between the treated and control groups was evaluated using log‐rank tests for survival curves. Data were represented as the mean ± SD for each experimental group. SPSS 13.0 software (SPSS Inc., Chicago, IL, USA) was utilised to analyse the experimental data. Statistical significance was defined as *p* < 0.05.

## 3 Results

### 3.1 Screening of Compounds Targeting ClpP in *S. aureus*

**FIGURE 1:**
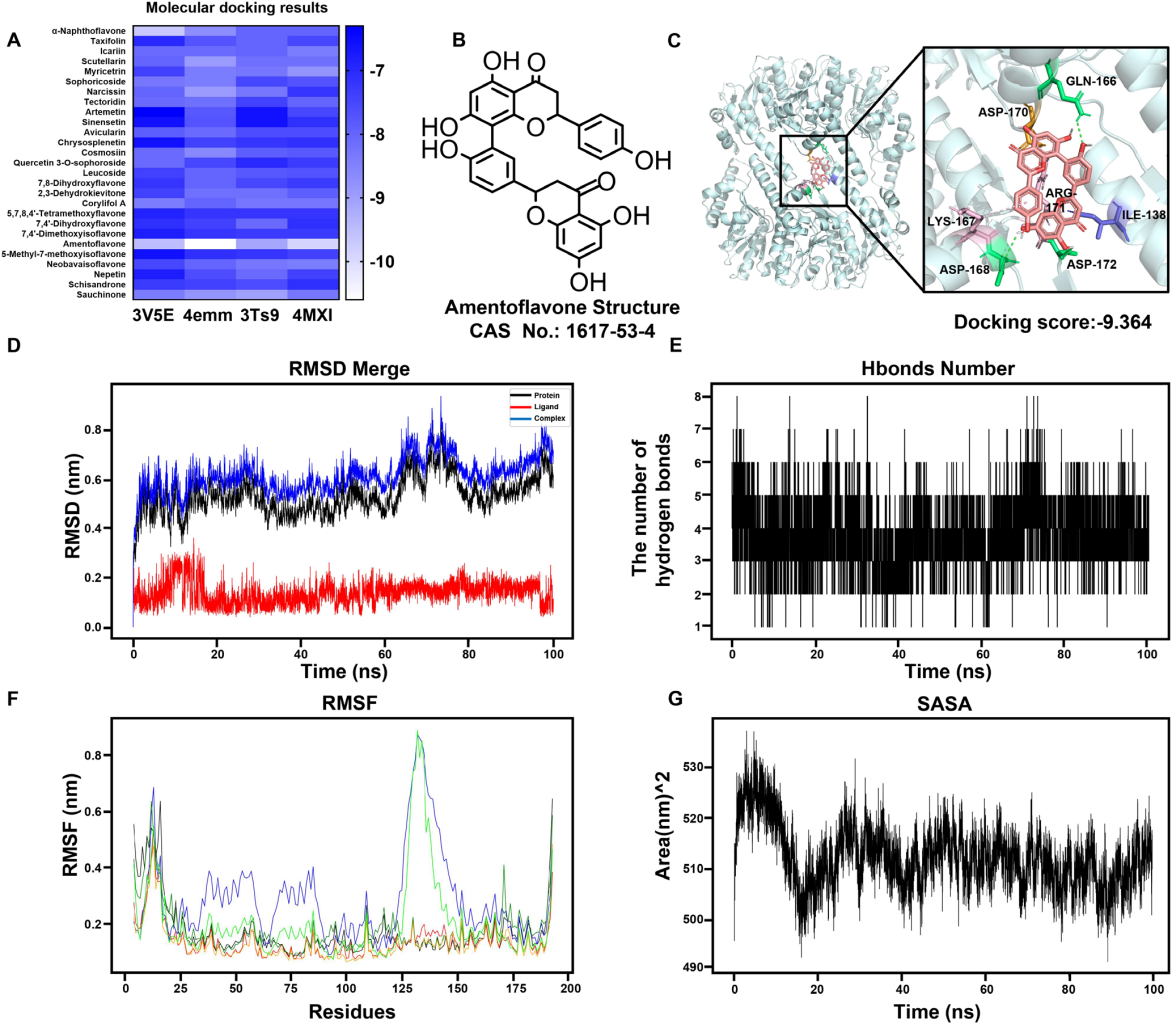
Molecular docking screening of flavonoid and glycoside natural compounds in our laboratory using ClpP as a target site. (A) Prediction of natural compounds by macromolecular screening with four ClpP conformations. (B) Structural formula and CAS number of AMF. (C) Autodock Vina was used to dock AMF and ClpP molecules, predicting the highest affinity AMF binding structure of ClpP. (D) RMSD of AMF, ClpP protein and their complex. (E) A number of hydrogen bonds between AMF and ClpP protein binding. (F) Deviation of ClpP protein subunits from the reference position. (G) The biomolecular surface area of ClpP protein subunits with AMF in a solvent.

The primary drug library of our laboratory was employed for molecular docking screening, utilising four structural models of ClpP in *S. aureus* . This assay revealed that the docking score between ClpP and AMF was comparatively lower than that with other compounds, prompting us to focus on AMF (Figure 1A,B). The docking sites were identified as ARG‐171, ASP‐170 and ASP‐172 (Figure 1C). To ensure the long‐term stability of the protein‐ligand complex, a kinetic model was employed for analysis. RMSD, a quantitative measure evaluating alterations in protein conformation, was calculated between the proteins and small molecules. The results indicated remarkable fluctuations in RMSD values during the initial 20 ns of simulation, followed by a gradual attainment of stability (Figure 1D). Overall, the RMSD values remained relatively consistent, suggesting a robust binding affinity between proteins and small molecules. Additionally, the Hbonds metric was employed to assess hydrogen bonds formed between proteins and small molecules. The analysis unveiled the formation of numerous hydrogen bonds between the protein and small molecule throughout the simulation, encompassing crucial residues and significant functional groups (Figure 1E). RMSF is a widely used metric to evaluate protein dynamics. The computed RMSF values between proteins and small molecules unveiled a revelation: the protein's RMSF value exhibited a remarkable decrease in the bound region, while experiencing an elevation in the non‐bound region (Figure 1F). This compelling evidence strongly suggests that the interaction with small molecules exerts a profound influence on the stability of the protein. In addition, SASA serves as a quantitative measure of protein surface area. We also calculated the SASA values between proteins and small molecules and demonstrated that prior to binding with the small molecule, the protein exhibited a higher SASA value which subsequently decreased upon binding (Figure 1G), indicating a reduction in the protein's exposed surface area due to interaction with the small molecule. Consequently, these findings imply that AMF may selectively target ClpP.

### 3.2 AMF Inhibits the Expression of Virulence Factors by Binding to ClpP and Inhibiting Its Activity

**FIGURE 2:**
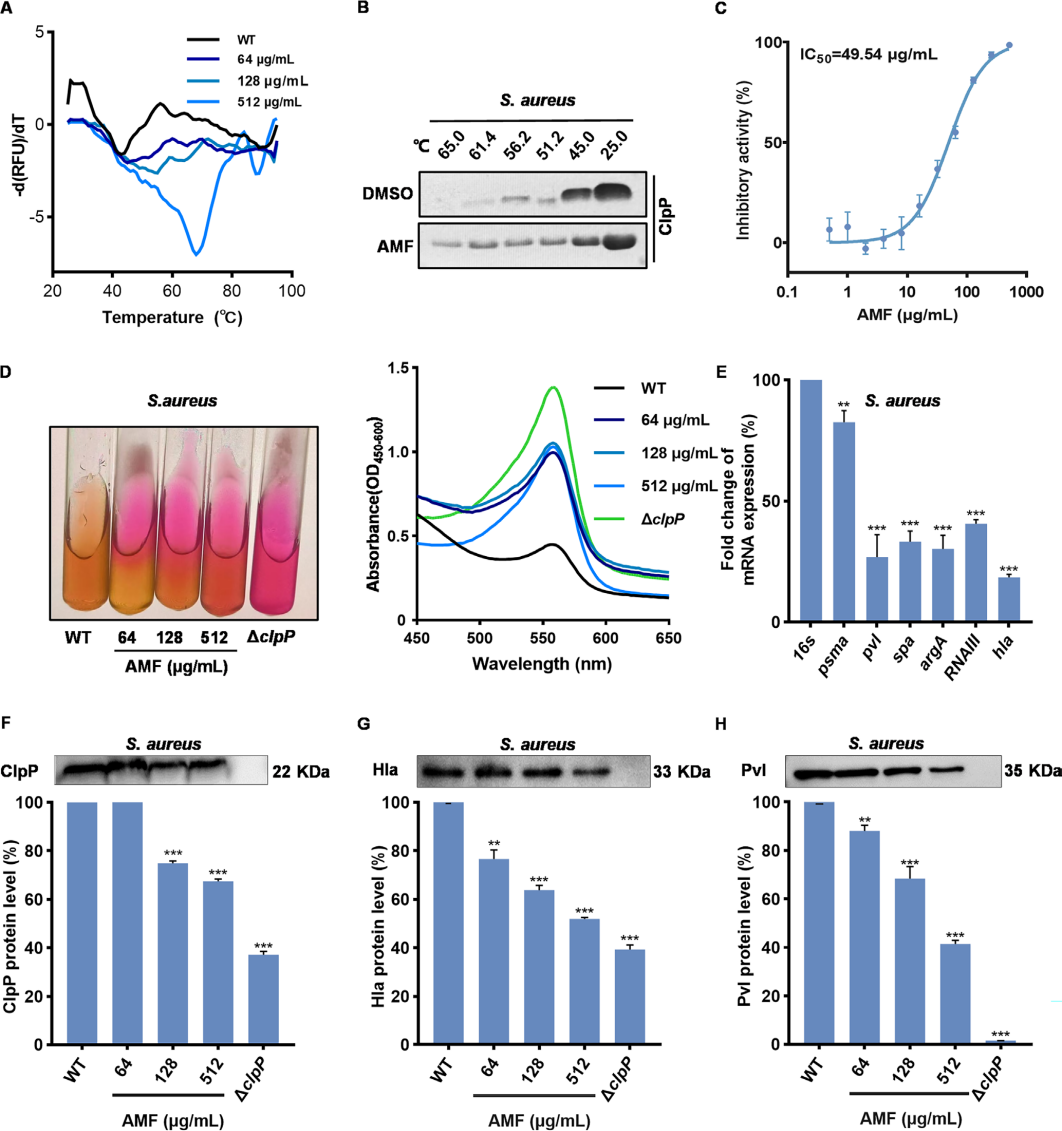
AMF binds to ClpP protein to reduce the expression of MRSA virulence factors. (A) Verification of the interaction between AMF and ClpP protein using TSA, detected ClpP thermal stability in the presence of AMF through fluorescence thermal stability assay. (B) CETSA verified the interaction between AMF and ClpP. SDS‐PAGE grayscale image of ClpP thermal stability in bacterial lysate after 60 min of reaction in DMSO dissolved AMF. (C) In fluorescence resonance energy transfer (FRET) experiments, AMF inhibited ClpP protein cleavage of the Suc‐LY‐AMC substrate at concentrations of 1 and 100 μM for ClpP and Suc‐LY‐AMC, respectively. The assay kit contains ClpP reaction buffer, purified ClpP protein (1 μM), various concentrations of AMF (0.5–512 μg/mL) and equal amounts of DMSO as negative controls. The emission wavelengths were set to 465 and 360 nm respectively, and the fluorescence intensity was measured with a microplate reader. (D) Urease medium of *Staphylococcus aureus* treated with different concentrations of AMF and its OD values at 450–650 wavelengths. (E) Transcript levels of the genes *agr*, *RNAIII*, *hla*, *pvl*, *psmα*, *spa* and *clpP* associated with exposure to an AMF concentration of 64 μg/mL were determined by RT‐qPCR. The reference gene used in the RT‐qPCR was *16 s* RNA. (F–H) Expression of ClpP, Hla and Pvl proteins in *S. aureus* treated with different concentrations (64–512 μg/mL) of AMF as measured by western blot. Significance was calculated by a two‐tailed *t*‐test: **p < 0.01 and ***p < 0.001. Repeat the experiment three times.

Subinhibitory concentrations were determined by MIC assay, and the subsequent series of ClpP‐related experiments were performed using this predetermined concentration as the basis. The thermal shift assay (TSA) is a highly effective technique for assessing protein‐ligand affinity, as it can accurately measure a wide range of affinities spanning from millimolar to picomolar levels within a single experiment [22]. Consequently, we employed TSA assays to ascertain the effects of AMF on the ClpP target. Our findings demonstrated that the addition of 64 μg/mL AMF resulted in a notable increase in the thermal stability of ClpP, with a *T*_m_ value reaching 44°C and an accompanying Δ*T*_m_ of 2°C. Furthermore, as the concentration of AMF increased, there was a corresponding augmentation in the magnitude of Δ*T*_*m*_. These observations provide compelling evidence for the significant enhancement of ClpP protein stability by AMF, thereby suggesting a direct interaction between AMF and ClpP protein (Figure 2A). The CETSA results, in line with the TSA findings, also revealed that AMF exerted a profound influence on enhancing the protein stability of ClpP. These aforementioned results further substantiated the binding affinity between AMF and ClpP (Figure 2B).

Subsequently, we utilised the ClpP‐specific fluorescent substrate Su‐LY‐AMC to assess the inhibitory impact of AMF on ClpP activity. In the presence of the intact fluorescent substrate, fluorescence resonance energy transfer between the fluorescent groups hinders the detection of fluorescence produced when ClpP recognises and hydrolyses the substrate peptide [23]. Upon separation of the fluorescent groups, fluorescence becomes detectable. Results showed that the addition of AMF led to a gradual decrease in fluorescence intensity, indicating a pronounced inhibitory effect on ClpP activity (Figure 2C).

Urease plays a pivotal role in the acid stress response system of *S. aureus* , contributing significantly to environmental adaptability, virulence and immune evasion [24]. Previous studies have demonstrated that ClpP exerts suppressive effects on urease production in *S. aureus* , as evidenced by the significant increase in urease production upon deletion or inhibition of ClpP [25]. The results of our assays investigating urease production in bacteria exposed to varying concentrations of AMF revealed a remarkable increase in the content of urease. As the concentration of AMF increased, the urease agar medium gradually transformed into red, resembling the Δ*clpP* group (Figure 2D). This strongly suggests that AMF may exert its influence on urease expression levels by modulating ClpP activity.

ClpP plays a crucial role in the biosynthesis of virulence factors in *S. aureus* . Hence, we employed quantitative real‐time PCR technology to investigate the impact of 512 μg/mL AMF on the mRNA expression of key virulence genes, including *spa*, *psm‐a*, *agrA*, *pvl*, *hla* and *RNAIII*. Results showed a significant down‐regulation of important virulence factors such as *hla*, *pvl*, *psm‐a* and spa in *S. aureus* ; notably, *hla* exhibited pronounced down‐regulation (Figure 2E). We further investigated the inhibition of AMF on the expression of ClpP, Hla, and PVL in *S. aureus* . Results indicated a gradual reduction in ClpP protein expression upon treatment with varying concentrations (64–512 μg/mL) of AMF, suggesting that AMF not only interacted with ClpP to impact its activity but also influenced its expression, thereby interfering with its function (Figure 2F). AMF also exhibited significant inhibitory effects on the expression of PVL and Hla protein, particularly at a high concentration of 512 μg/mL, resulting in a remarkable decrease in PVL expression by 41.50% ± 12.58% and Hla protein expression by 51.72% ± 16.12%. Notably, the expression of Hla and PVL proteins was significantly reduced in the Δ*clpP* strain (Figure 2G,H). In summary, our findings demonstrated that AMF exerted an inhibitory effect on the expression of key virulence factors, including Hla and PVL, through its interaction with ClpP and subsequent inhibition of its activity.

### 3.3 The Inhibition Effect of AMF on Biofilm Formation and Haemolysis Effect of *S. aureus*

**FIGURE 3:**
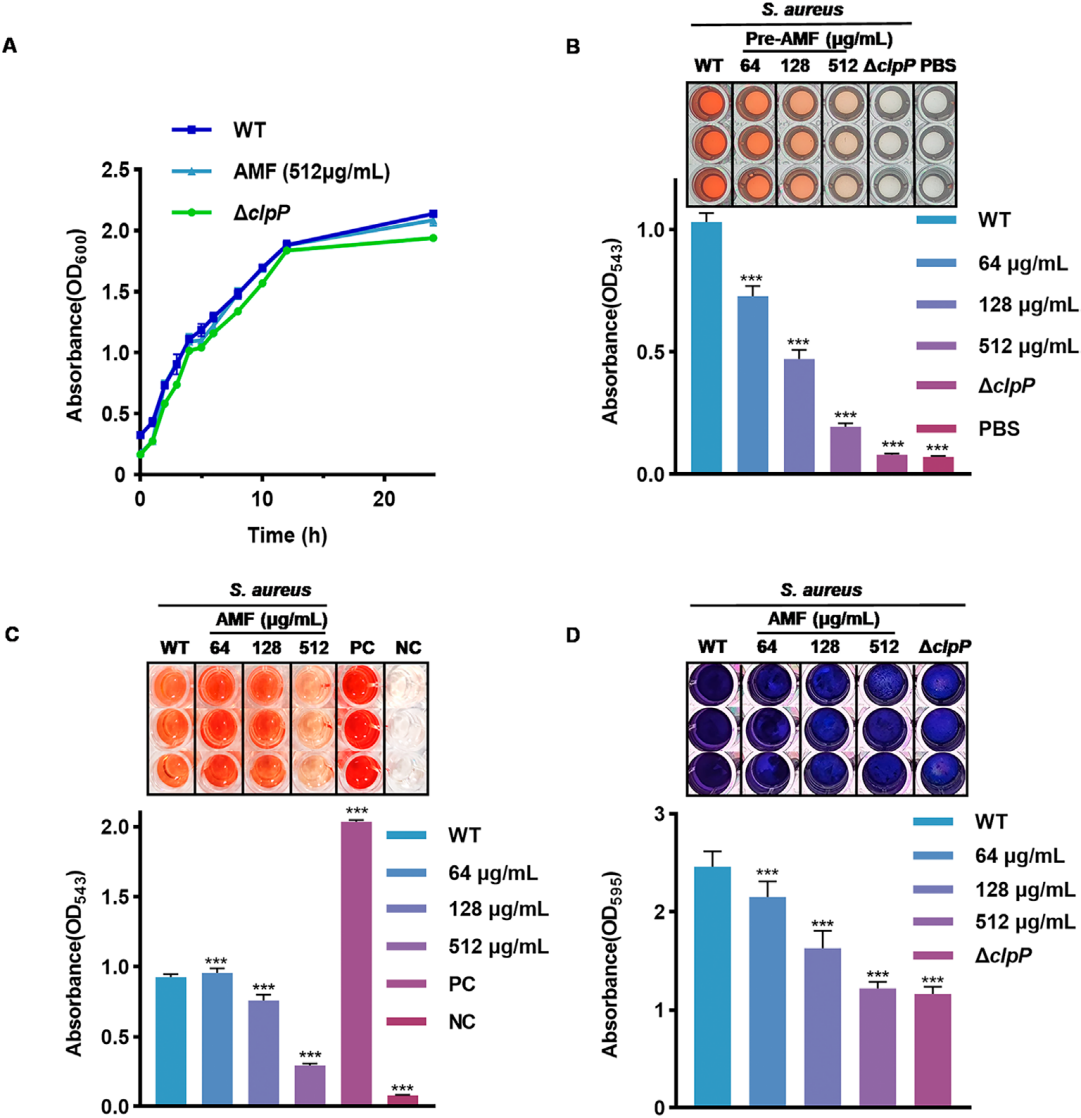
The inhibition effect of AMF on biofilm formation and haemolysis effect of *Staphylococcus aureus.* (A) Measurement of OD values of 512 μg/mL AMF‐treated *S. aureus* over 24 h. (B) The haemolytic effect of *S. aureus* pretreated with different concentrations (64–512 μg/mL) of AMF on rabbit erythrocytes was observed and OD values were measured. (C) To observe the effect of AMF on the haemolytic ability of *S. aureus* when destroying rabbit red blood cells, various concentrations of AMF (64–512 μg/mL) were added, and the OD value was measured. (D) The inhibition of biofilm formation in *S. aureus* by AMF at different concentrations (64–512 μg/mL) was observed and OD values were measured. Significance was calculated by a two‐tailed *T*‐test: **p < 0.01 and *** *p* < 0.001. Repeat the experiment three times.

Natural products hold great potential for the development of antivirals with multiple mechanisms to combat pathogens and reduce bacterial virulence [26]. Our study mentioned above that AMF bound to ClpP and exerted inhibitory effects on its activity by affecting its expression. The bacterial growth curve experiment revealed that AMF had no significant impact on bacterial growth (Figure 3A). Notably, the concentration of AMF used in our experiments is much lower than the MIC of the drug, suggesting that AMF controls *S. aureus* infection by reducing MRSA virulence rather than inhibiting bacterial growth. The haemolysis test demonstrated that both pretreating MRSA with AMF and the addition of AMF during the haemolysis process effectively mitigated the haemolysis of *S. aureus* on rabbit red blood cells (Figure 3B,C). Additionally, our experiments also indicated that AMF could impede the biofilm formation ability of *S. aureus* (Figure 3D). Based on the above results, we confirmed that AMF possesses the capability to attenuate the virulence of MRSA and impede its biofilm formation ability, thereby providing cellular protection against infection.

### 3.4 AMF Protects *A549* Cells From Invasion of Methicillin‐Resistant *S. aureus*

**FIGURE 4:**
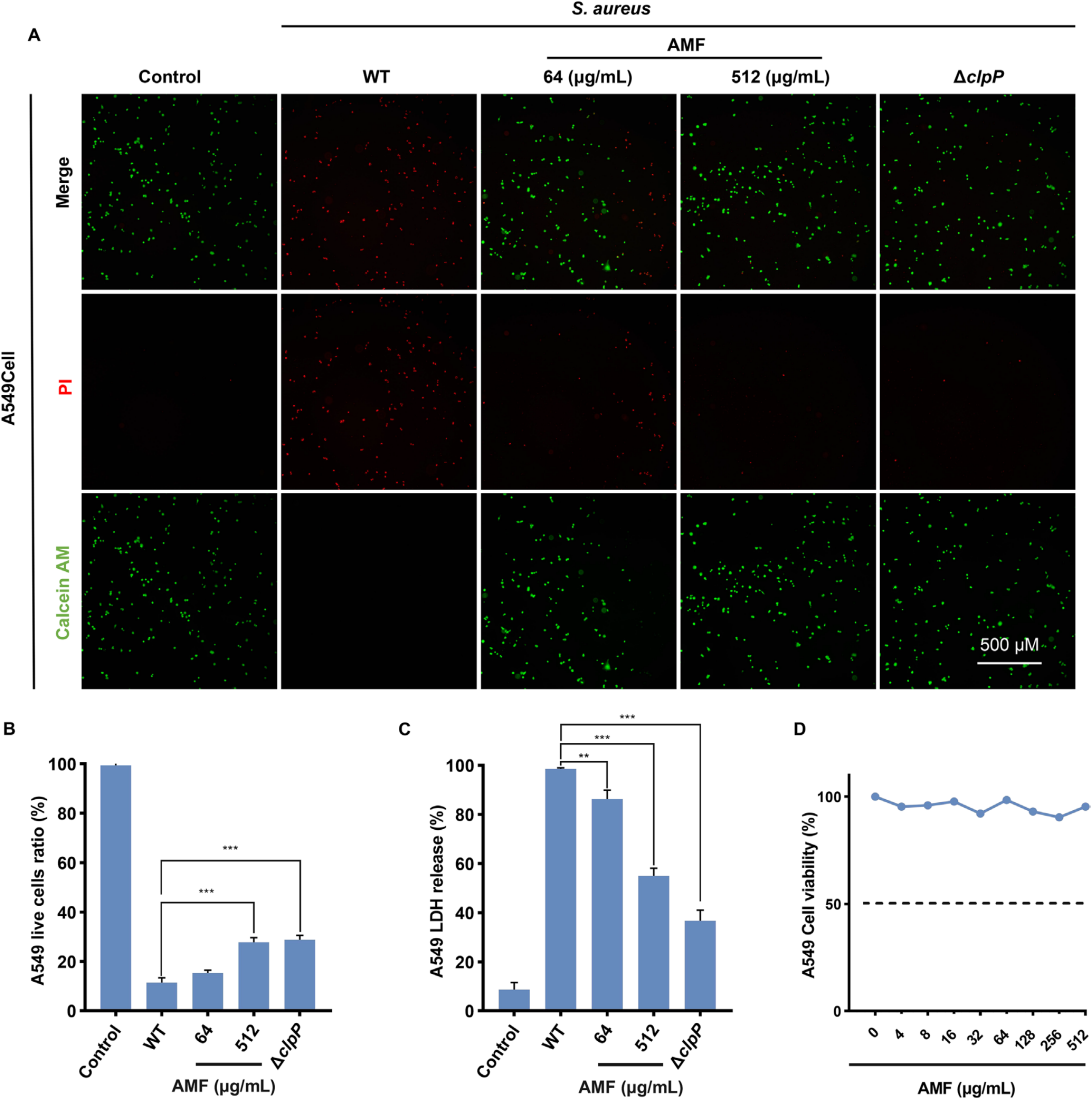
AMF protects A549 cells from the invasion of MRSA (A) Fluorescence imaging observation of calcium xanthophyll AM/PI staining of A549 cells under different conditions, red for dead cells and green for live cells. (B) A549 cells were treated with different concentrations of AMF after *Staphylococcus aureus* infestation. Live and dead A549 cells under different conditions were counted by Image.J. (C) A549 cells infected with *S. aureus* were treated with different concentrations of AMF, and the ratio of LDH release from A549 cells was calculated. (D) Detection of AMF toxicity to common A549 cells. Significance was determined by a two‐tailed *t*‐test: **p < 0.01, ***p < 0.001. The experiment was repeated three times.

MRSA is a common cause of hospital‐acquired pneumonia [27], with studies showing an increasing proportion of MRSA in patients with infectious pneumonia leading to higher morbidity and mortality rates among hospitalised individuals [28]. In this study, our investigation aimed to determine the potential of AMF in preventing MRSA infection in A549 cells. A549 cells were separately exposed to equal amounts of MRSA and subjected to different treatments. Cell cytotoxic activity was assessed using Live & Dead Calcein AM, PI assay kits. Results revealed an increase of viable cells (green fluorescence) in the visual field post‐treatment with AMF compared to the WT group, and the effect of the 512 μg/mL AMF treatment group was equivalent to the knockout of the ClpP group (Figure 4A,B). The findings suggested that AMF has the potential to decrease infection and damage to A549 cells caused by *S. aureus* . To validate these results, the viability of A549 cells was assessed using the LDH method. The data indicated that the LDH release rate of A549 cells decreased following treatment with AMF (512 μg/mL), suggesting a protective effect against *S. aureus* in A549 cells (Figure 4C). When the MTT method was employed to evaluate the cytotoxicity of AMF on A549 cells, experimental results showed that AMF did not exert a significant impact on A549 cell proliferation and thus could be considered safe for medical application (Figure 4D). In summary, our study provided evidence supporting the efficacy of AMF in controlling MRSA‐induced infections and its protective effect on cells.

### 3.5 Efficacy of AMF in Combating Lung Infection Induced by *S. aureus*

**FIGURE 5:**
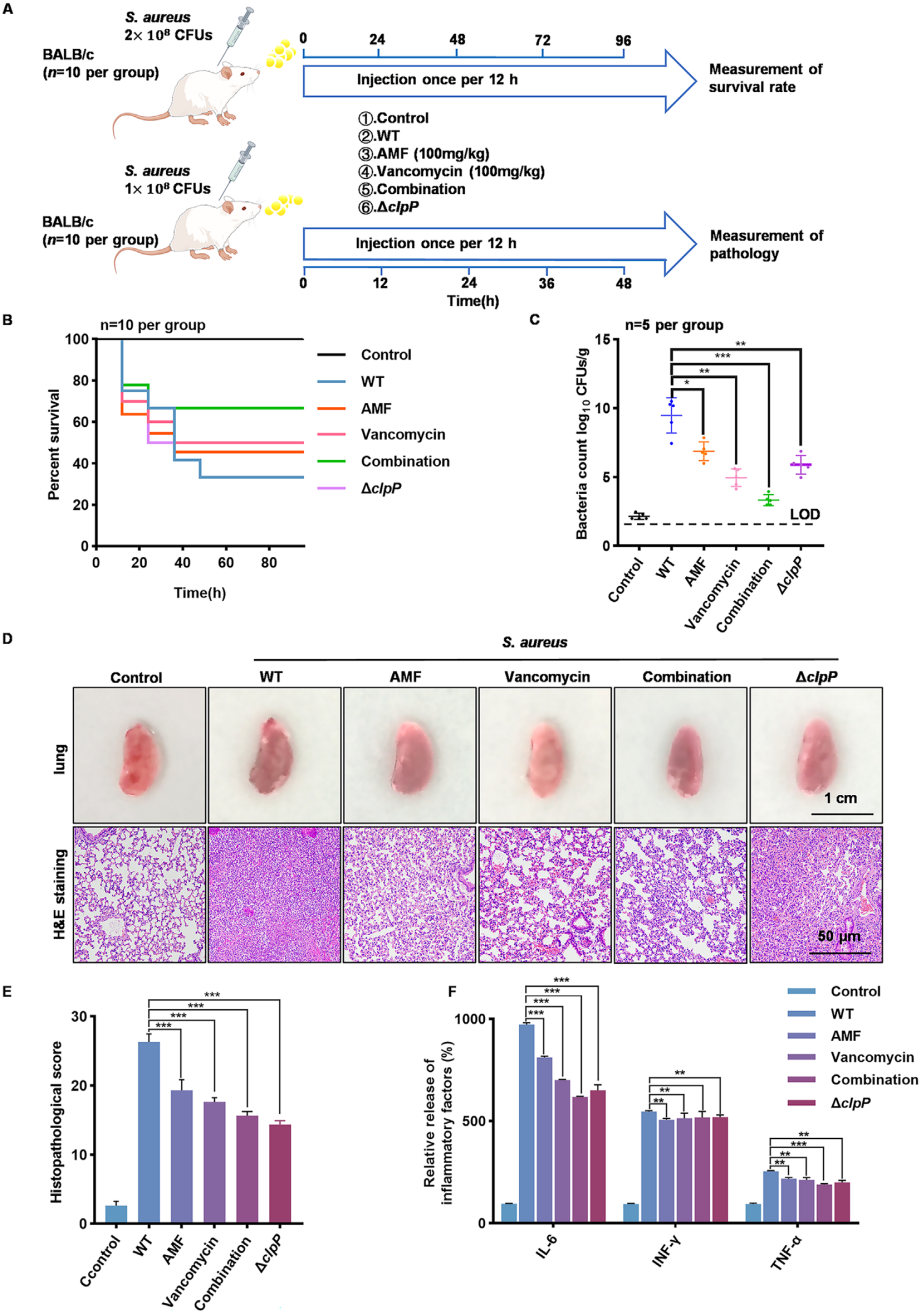
Efficacy of AMF in combating lung infection induced by *Staphylococcus aureus.* (A) Experimental flow chart of using USA300 to invade mice to form MRSA pneumonia, in which the mice were pre‐anaesthetised with ether and divided into different groups. In the livability experiment, all mice were intranasally instilled with 2 × 10^8^ CFUs of MRSA. Mouse deaths were recorded every 12 h for a total of 96 h, and the overall livability was summarised. In pathological studies, all mice were intranasally instilled with 1 × 10^8^ CFUs of USA300. The process conditions were based on the livability experiment, and changes in bacterial load in the lungs were detected after 48 h. The results were used to determine the livability of the mice. (B) Results of the livability of each group of mice in 96 h. (C) Results of bacterial load in the lungs of mice in each group under limit of detection. (D) Gross and histopathological examination of lung tissue. Scale bar, 50 μ m. (E) Lung histopathology scoring. Lung injury status was assessed by local haemorrhage, inflammatory cells, embedded areas, oedema and bronchiolar exudate in H&E stained sections. Pathology scoring of mouse lungs was performed by summarising all indicators to obtain a score from 0 (not affected) to 26 (very affected) (*n* = 5). (F) Contents of TNF‐α, IFN‐γ and IL‐6 in alveolar lavage fluid of mice under different conditions. Significance was determined by a two‐tailed *t*‐test: **p < 0.01, ***p < 0.001. The experiment was repeated three times.

*S. aureus* infection leads to severe lung inflammation [29], increasing morbidity and mortality. To assess the inhibitory effect of AMF on *S. aureus* ‐induced infections, we established a pneumonia model by intranasal infection in *BALB/c* mice. Each mouse received 2 × 10^8^ CFU of *S. aureus* intranasally and was randomised into six groups, including the blank control group, the *S. aureus* infection group, Δ*clpP* group, 100 mg/kg AMF group, vancomycin group and combination treatment group (Figure 5A). Consistent with previous studies, mice infected with *S. aureus* exhibited a significantly diminished survival rate of only 30% within 96 h. However, both the administration of AMF treatment and the knockout of ClpP groups remarkably augmented the survival rate in mice, with an even more pronounced effect observed when AMF was combined with vancomycin (Figure 5B). Consistently, compared with the WT group, the bacterial load in the lungs of mice in the AMF‐treated group and the knockout ClpP group was significantly lower, and the effect of AMF in combination with vancomycin was more pronounced (Figure 5C). It indicated the potential ability of AMF in combating lung infection caused by *S. aureus* and highlighted the crucial role of ClpP in regulating the virulence of *S. aureus* . Furthermore, this phenomenon illustrates the combined effect of AMF and vancomycin. We also examined the lung tissue damage in mice. The results showed that compared with the control group, the lung tissue infected by *S. aureus* was severely congested, darker in colour, lost elasticity and had obvious turbid tissue fluid in the alveolar cavity. The AMF treatment, however, exhibited remarkable efficacy in alleviating lung tissue congestion, restoring elasticity and mitigating inflammatory cell infiltration. These findings strongly suggested that AMF held great potential in ameliorating lung infections (Figure 5D,E). Compared to the MRSA infection group, the levels of various inflammatory factors (IL‐6, INF‐γ, TNF‐α) in the alveolar lavage fluid of the treatment group showed varying degrees of reduction. Notably, IL‐6 exhibited the most significant decrease, with the combination treatment group demonstrating the most favourable outcome (Figure 5F). Therefore, the above experiments suggested a protective effect of AMF against MRSA fatal pneumonia.

## 4 Discussion

Antivirulence strategies represent a promising approach in combating bacterial resistance [30]. At their core, these innovative approaches aim to undermine the virulence and pathogenicity of bacteria by precisely targeting the pivotal mechanisms involved in bacterial infections [31]. Such mechanisms, encompassing host invasion, colonisation, immune evasion and toxin secretion, are crucial for bacterial pathogenesis [32]. MRSA could present clinically as asymptomatic colonisation of the nasal mucosa [33], mild skin and soft tissue infections [34] or severe invasive diseases with high mortality rates [35], all of which are linked to the diverse virulence factors of the bacteria. ClpP, a key player in MRSA pathogenesis, exerts direct and indirect control over the majority of its virulence factors [36]. For instance, ClpP influences bacterial pH balance, host cell invasion, red and white blood cell destruction and immune evasion [37]. Targeting ClpP proves to be a more efficacious strategy compared to tackling individual virulence factors, making it a primary focus in the development of antivirulence therapies. While both the activation [38] and inhibition [39] of ClpP could culminate in bacterial death, the quest for a stable and potent ClpP agonist has thus far remained an unattained goal [40]. Consequently, the prevailing emphasis lies in the inhibition of ClpP. In recent years, there has been extensive research on anti‐infective drugs targeting MRSA. These studies primarily investigate the impact of drugs on the internal structural proteins of MRSA, such as inhibiting bacterial cell wall formation [41], regulating cell membrane function [42] and inhibiting DNA and RNA synthesis [43]. Unlike previous studies, our research specifically targeted the inhibition of ClpP activity, thereby reducing the virulence of MRSA.

AMF, the premier natural flavone extracted from oak trees, exhibits unique biological activities such as antioxidant, anticancer, antibacterial and antiviral properties. In 2008, Bottcher and Sieber identified a group of β‐lactones that function as ClpP inhibitors in *S. aureus* and *Listeria monocytogenes* [44]. However, the hydrolysis of cyclic ketones diminishes plasma stability and results in poor selectivity. The modified phenyl ester AV170 enhances plasma stability and pharmacokinetics but decreases antitoxicity. Subsequently, boron‐containing compounds were recognised as potential ClpP inhibitors for treating tuberculosis; however, these compounds are expensive, exhibit short half‐lives and display poor pharmacokinetics [45]. Natural product protein inhibitors offer several advantages including potent biological activity, diverse structures, low toxicity, minimal drug resistance and sustainability, making them invaluable assets in the realm of drug development [46]. Therefore, it may prove more prudent to seek out natural compounds capable of efficaciously inhibiting ClpP activity.

In this study, we investigated the different configurations of ClpP in MRSA and conducted molecular docking experiments to screen for potential drug candidates. Our findings revealed that AMF exhibited strong interactions with ARG‐171, ASP‐170 and ASP‐172 of ClpP. Subsequent molecular dynamics simulations and thermal shift experiments also suggested that the interaction between AMF and ClpP was stable. FRET experiments have demonstrated that AMF was capable of binding to the ClpP protein. In vitro experiments revealed that the natural compound AMF significantly impeded the destruction of red blood cells by *S. aureus* without affecting bacterial growth. Unlike other ClpP inhibitors, AMF not only inhibited ClpP activity but also countered the damaging effects of α‐hemolysin secreted by *S. aureus* on red blood cells In vitro.

**FIGURE 6:**
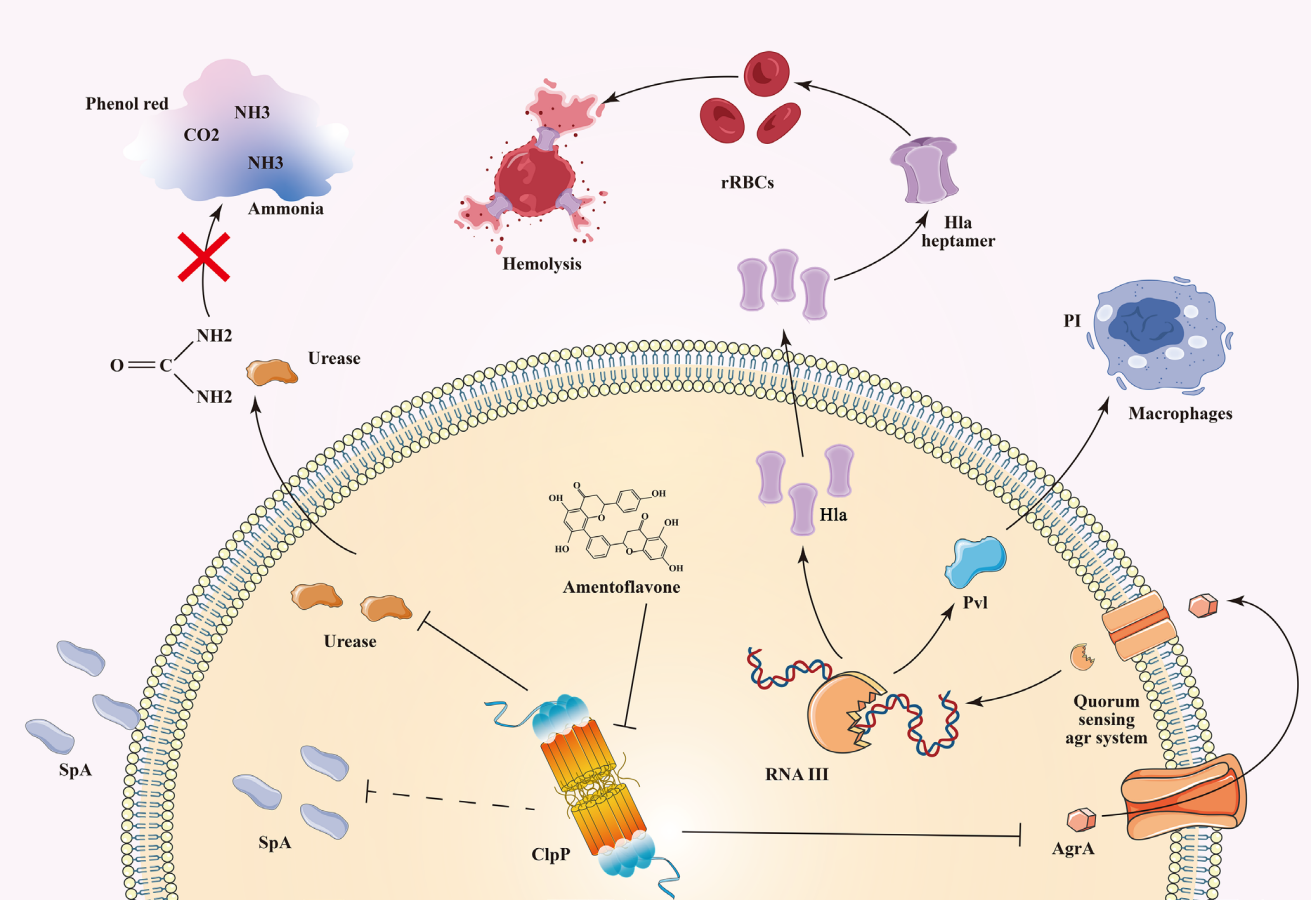
AMF targets ClpP to affect the expression of multiple virulence factors, leading to a decrease in the pathogenicity of MRSA (A) AMF binds to the ClpP protein to inhibit its activity, activating the Agr sensing system, which in turn results in the accumulation of functional peptides outside the bacterium via channel proteins. The AgrC protein regulates the RNAIII of the AgrA protein in the bacterium, reducing the production of Hla and PVL. Hla can be secreted from *Staphylococcus aureus* to form heptamers that adsorb to the surface of erythrocytes, destroying them. PVL can bind to leukocytes to disrupt leukocyte homeostasis and cause leukocyte death. The ClpP protein has an inverse regulatory effect on urease, inhibiting urease production. It inhibits urease to produce urease inhibitors, which are secreted outside the bacterium to break down the substrate urea into ammonia and turn the medium red. Indirect regulation of Spa proteins.

AMF showcases remarkable efficacy in enhancing the resistance of A549 cells against *S. aureus* invasion by reducing cellular LDH release rate, minimising cell damage and enhancing cell survival rate. Furthermore, AMF exhibits the ability to lower bacterial load and virulence in lung tissue and regulate lung bleeding, swelling and tissue fluid exudation, ultimately improving the survival status and rate of mice. When administered in combination with vancomycin, AMF shows superior outcomes, significantly decreasing various pathological indicators compared to vancomycin monotherapy. Overall, AMF mitigates the virulence of *S. aureus* through multiple pathways, offering comprehensive protection to mice (Figure 6).

In conclusion, the exploration and development of natural product protein inhibitors hold promise for the discovery of novel drugs and the enhancement of treatment outcomes for various diseases. Our study suggested that AMF holds promise as an effective antivirulence agent that could be utilised in combination with antibiotics for treating infections caused by MRSA.

## Author Contributions

All authors contributed to the study conception and design. Teri Gele: conceptualisation (lead); writing – original draft (lead). Xiangri Kong: writing – review and editing. Qiuyue Zhang: supervision. Wu Song: project administration. Junpeng Guo: software. Paizati Hamidi: validation (equal). Kulishasi Mani: validation (equal). Shaoyu Han: formal analysis (equal). Xuan Zhao: formal analysis (equal). Jingwen Chen: formal analysis (equal). Chi Zhang: data curation (lead). Abduldayeva Aigul Abduldayevna: resources. Qingjie Li: funding acquisition (lead).

## Ethics Statement

The animal experiments in this report fully followed the International Guidelines for Biomedical Research Involving Animals issued by the Council for International Organisations of Medical Sciences (CIOMS) and the guidelines of ARRIVE, and complied with the relevant regulations for the use of laboratory animals. The animal experimental procedures complied with the standards of the Laboratory Animal Ethics Committee of Changchun University of Chinese Medicine, with the ethical approval number 2022541.

## Conflicts of Interest

The authors declare no conflicts of interest.

## Data Availability

Data concerning the results obtained or analysed during this study can be obtained by contacting the author of this article upon reasonable request.
